# Clinical pharmacists’ interventions across German hospitals: results from a repetitive cross-sectional study

**DOI:** 10.1007/s11096-021-01313-3

**Published:** 2021-08-17

**Authors:** Claudia Langebrake, Carina Hohmann, Susanne Lezius, Michael Lueb, Gesine Picksak, Wencke Walter, Sandra Kaden, Heike Hilgarth, Angela Ihbe-Heffinger, Katja Leichenberg

**Affiliations:** 1 Hospital Pharmacy, University Medical Center Hamburg-Eppendorf, Martinistraße 52, 20246 Hamburg, Germany; 2 Department of Stem Cell Transplantation, University Medical Center Hamburg-Eppendorf, Hamburg, Germany; 3 Department of Pharmacy, Klinikum Fulda gAG, Fulda, Germany; 4 Institute of Medical Biometry and Epidemiology, University Medical Center Hamburg-Eppendorf, Hamburg, Germany; 5 Department of Pharmacy, Klinikum Bielefeld Gem. GmbH, Bielefeld, Germany; 6 Pharmacy, Hannover Medical School, Hannover, Germany; 7 Department of Pharmacy, University Hospital Dresden, Dresden, Germany; 8 Hospital Pharmacy, Klinikum Starnberg GmbH, Academic Teaching Hospital of the Ludwig-Maximilians University, Starnberg, Germany; 9 Department of Pharmacy, University Hospital Jena, Jena, Germany; 10 Department of Intensive Care Medicine, University Medical Center Hamburg-Eppendorf, Hamburg, Germany

**Keywords:** Pharmacists’ interventions, Medication therapy management, Medication errors, Drug related problem, Pharmacy service, hospital

## Abstract

*Background* Pharmacists’ interventions (PI) are suitable to improve medication safety and optimise patient outcome. However, in Germany, clinical pharmacy services are not yet available nationwide. *Aim* To gain prospective data on the extent and the composition of routine PI with special focus on intervention rates among German hospital pharmacists during two intervention weeks. *Methods* Within a repetitive cross-sectional study, clinical pharmacists documented all PIs on five days during a one-month period (intervention week) in 2017 and 2019 using the validated online-database ADKA-DokuPIK. Additionally, data regarding the supply structure/level of medical care, the extent of clinical pharmacy services and their professional experience were collected. All data were anonymised before analysis. *Results* In total, 2,282 PI from 62 pharmacists (2017) and 2578 PI from 52 pharmacists (2019) were entered. Intervention rate increased from 27.5 PI/100 patient days in 2017 to 38.5 PI/100 patient days in 2019 (*p* = 0.0097). Frequency of clinical pharmacy services on a daily basis significantly increased from 60% (2017) to 83% (2019). Reasons for PIs from the categories “drugs” (e.g. indication, choice, documentation/transcription) and “dose” were most common in both intervention weeks. The vast majority of underlying medication errors in both intervention weeks were categorised as “error, no harm” (80.3 vs. 78.6%), while the proportion of errors which did not reach the patient, doubled to 39.8% in IW-2019. *Conclusion* Regular and daily clinical pharmacy services become more established in Germany and clinical pharmacists are increasingly involved in solving drug related problems proactively and early during the medication management process.

## Impacts on practice

- The frequency of clinical pharmacy services in terms of proactive medication management in German hospitals increased between 2017 and 2019.
- Intervention rate to solve drug-related problems by German clinical pharmacists is increasing to more than one out of three patients.
- Involvement of clinical pharmacists early in the medication management process improves patient safety by detection and avoidance of medication errors, before reaching the patient.

## Introduction

Pharmacists’ interventions (PI) have been shown to positively influence clinical outcome of hospital inpatients [1–5]. Although clinical pharmacy services (CPS) are well established in many countries worldwide, Europe, and especially Germany is lacking behind [6–8]. Hospital pharmacists in around half of the German hospitals work routinely as part of a multidisciplinary team (EU mean: 47.8%, UK: > 90%), while in less than half of the German hospitals all prescriptions are reviewed and validated as soon as possible (EU mean: 54.9%, UK: 100%) [9]. During the last years, the topic of CPS and medication management has gained a greater focus of attention in Germany. According to a recent national survey, at least 22% of hospital pharmacies in Germany provide some form of CPS with a wide variation between hospitals [10].

DokuPIK is a national anonymous self-reported online documentation system, hosted by the German Association of Hospital Pharmacists (ADKA) for the voluntary documentation of medication errors (ME) and PI. A detailed description has been published elsewhere [11, 12], in brief basic anonymous patient data, classification of drugs using the World Health Organisation Anatomical Therapeutic Chemical classification (ATC) and hierarchical classification of reason, resulting actions, acceptance and severity of the underlying ME according the National Coordinating Council for Medication Error Reporting and Prevention (NCC MERP) [13] may be entered. With regard to DokuPIK a PI is defined as “any communication/action solving and/or avoiding drug related problems (DRPs)” and includes the “management of existing DRPs as well as any proactive approach avoiding potential DRPs within the medication use process” [14]. Recently, the categorisation quality of hospital PI using DokuPIK has been validated with a high level of agreement and a good specificity as well as positive and negative predictive value of 90%, despite the allowance of multiple choices [11].

In a previous study, we described the scope of clinical pharmacists’ involvement in patient care in daily clinical practice and demonstrated the usefulness and importance of proactive PI in the prevention of hazards and risks for hospital inpatients [14]. However, data concerning the extent of PI and the intervention rates in German hospitals and their potential changes—especially against the background of efforts to strengthen the professional role of clinical pharmacists during the last years—are missing.

### Aim of the study

The aim of our repetitive cross-sectional study was to gain prospective data on the extent and the composition of routine PI in German hospitals for a five working day scenario and to identify changes over time. We were especially interested in describing intervention rates on a population basis in terms of performed medication analyses, that might further be used as a quality indicator for CPS.

### Ethics approval

Our study does not require an ethics approval, as it is classified as quality assurance for documentation of PI in daily routine. All participating pharmacists voluntarily provided their data in the survey and gave consent to analyse and publish the results. All PI datasets in DokuPIK only contain anonymous patient data.

## Methods

All DokuPIK users were invited by e-mail to completely document all PIs, which were performed on five days during a one-month period (February 1st to 28th 2017 and November 1st to 30th, 2019, respectively). Furthermore, the users were asked to fill out an online survey using the socisurvey portal (https://www.soscisurvey.de/) with data regarding the supply structure/level of medical care, the extent of the CPS and their professional experience. DokuPIK users who participated in the DokuPIK intervention week (IW) flagged the datasets that were supposed to be included in the analysis and gave their consent to pseudonymise these datasets in terms of the user, who entered the data. Patient data remained completely anonymous. Pseudonymised data from the surveys were combined with the pseudonymised data sets from DokuPIK and anonymised before analysis.

All datasets of the DokuPIK IW 2017 were reviewed by at least one senior CP concerning data consistency. If one senior CP marked a dataset as not to fulfil the definition of a PI according to DokuPIK, the respective dataset was reviewed by five senior CP (all members of the ADKA special interest group “pharmacists’ interventions”) and a consensus decision was taken by majority. The same procedure was employed for those datasets, where the underlying ME was categorised as “E” or higher according to NCC-MERP [13]. For the DokuPIK IW 2019 it had been decided not to undertake the check for data consistency, because less than one percent of the datasets of the DokuPIK IW 2017 (22/2313) did not fulfil the definition of a PI and only nine duplicates were found.

“Patient days” were defined as the sum of days with patients receiving a medication analysis by the CP during a period of five working days. For example, if one CP performs medication analyses on a daily basis and sees 20 patients twice a week and 30 patients three times a week, the patient days would sum up to 130 (= 2 × 20 + 3 × 30), while the medication of an individual patient might have been analysed more than once during the week. For each participating pharmacist, the individual rates of performed PI per reported patient days (intervention rate) were calculated, e.g. if a pharmacist carried out 52 PI per week and had 130 patient days, the rate is 40/100 patient days.

Data were analysed anonymously using Excel (Version 2013, Microsoft) and SPSS (Version 22, IBM). Data that had been entered as “not known” in DokuPIK for the characterisation of the patient were considered as missing values in all analyses. For group comparisons of categorial variables, Chi-square or Fisher’s exact tests were used as appropriate. Continuous (but skewed) variables were analysed using Mann–Whitney-U-Test. To model the associations of the rate of interventions with number of interventions and professional experience, negative binominal regression was used and results are presented as incident rate ratios (IRR) with corresponding 95% confidence intervals (CI) and *p*-values. A *p*-value of < 0.05 was defined as statistically significant. No adjustment for multiplicity was performed, due to the explorative character of the analyses.

## Results

From the DokuPIK intervention week 2017 (IW-2017) 2282 datasets from 62 CP (out of 29 different hospitals) and from the DokuPIK intervention week 2019 (IW-2019) 2578 datasets from 52 users (out of 20 different hospitals) could be included in the final analysis.

A comprehensive overview of the participants’ characteristics is given in Table 1. Considerable changes towards a higher frequency of CPS were detectable in IW-2019 (*p* = 0.022), while the number and distribution of patient days with medication analysis (per week and pharmacist) were comparable.

**Table 1:** Characteristics of participants

	IW-2017 (n = 62)	IW-2019 (n = 52)	*p*-value
**Types of hospitals [number (%)]**			0.463
University hospital	34 (54.9%)	35 (67.3%)	
Maximum care hospitals	11 (17.7%)	7 (13.5%)	
Hospital of full medical care/specialist hospital	8 (12.9%)	3 (5.8%)	
General hospitals	9 (14.5%)	7 (13.5%)	
**Professional experience of participating pharmacists [Median (range), years]**			
Hospital pharmacist	9 (1.5–35)	9.5 (0.5–32)	0.984
Expert clinical pharmacist according to further training regulations	3 (0–30)	1.5 (0–25)	0.241
Clinical pharmaceutical services	6 (0.5–34)	6 (1–19)	0.451
**Frequency of clinical pharmaceutical services [number (%)]**			0.022
Daily	37 (59.7%)	43 (82.7%)	
2–3-times per week	14 (22.6%)	2 (3.8%)	
Once weekly	10 (16.1%)	6 (11.5%)	
Less than once weekly	1 (1.6%)	1 (1.9%)	
**Patient days per week [Median (IQR; range)]**	97 (40–211; 4–1000)	85 (30–158; 2–1500)	0.444
**Frequency of the use of ADKA DokuPIK [number]**			0.499
Daily	2 (3.2%)	3 (5.8%)	
2–3 times per week	8 (12.9%)	4 (7.7%)	
Once weekly	8 (12.9%)	6 (11.5%)	
Less than once weekly	14 (22.6%)	7 (13.5%)	
In the context of projects	30 (48.4%)	32 (61.5%)	

Patient days are defined as the sum of all patients seen by the CP during a period of five working days

*IQR* inter-quartile range

### Intervention rates

**Fig. 1:**
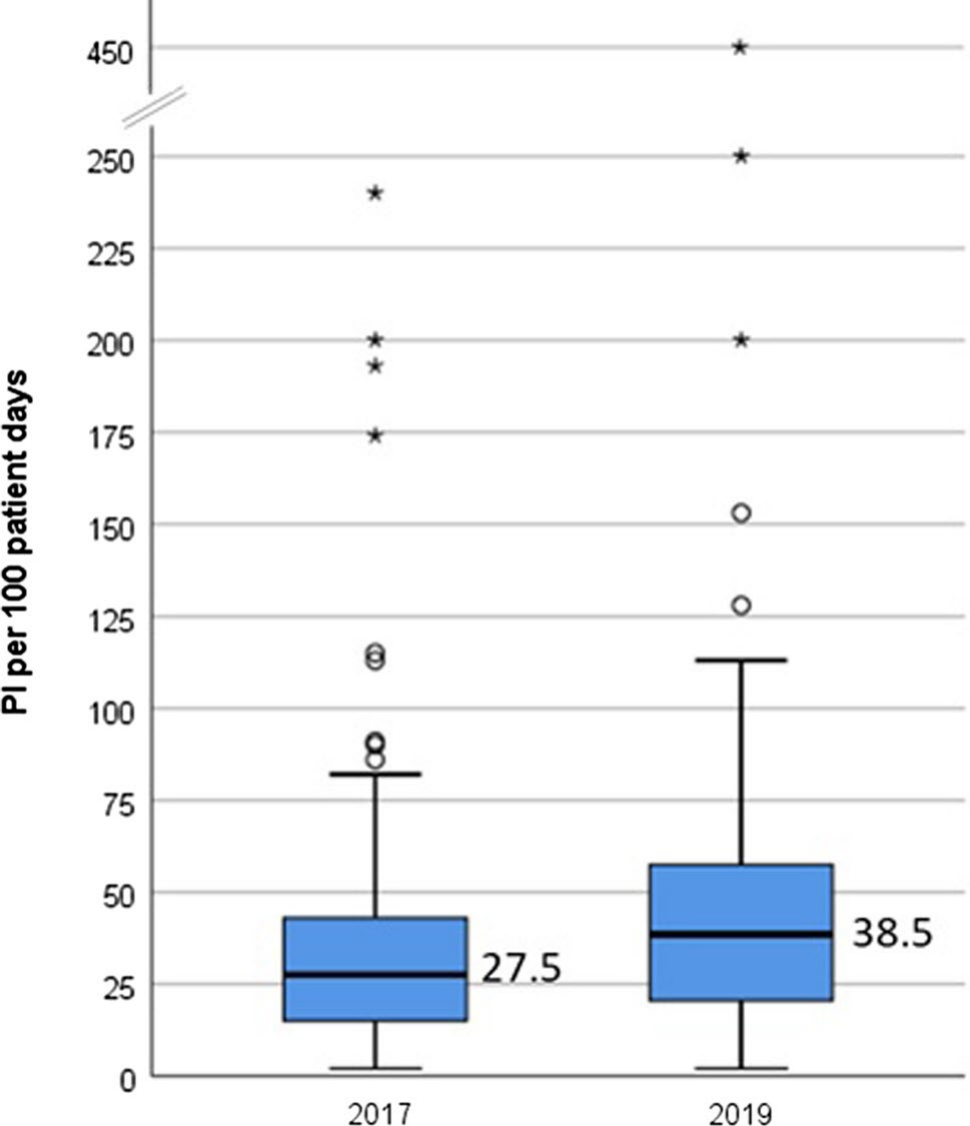
Intervention rates, expressed as PI per 100 patient days in IW-2017 and IW-2019 (boxes represent the 25th to 75th percentiles, whiskers: lowest and highest non-extrem values, circles: mild outliers, asterixes: extreme outliers)

The absolute numbers of documented PI per pharmacist per week were similar in IW-2017 and IW-2019 with a median of 22.5 (IQR: 11.5 to 49.5) and 23.5 (IQR: 10.8 to 51.3), respectively. However, relating the PI to patient days, there was a strong increase from 27.5 (IQR: 14.8 to 43.3) PI/100 patient days in IW-2017 to 38.5 (20.3 to 59.3) PI/100 patient days in IW-2019, as shown in Fig. 1. Although the differences are not statistically significant, there is a trend towards higher values in IW-2019 (*p* = 0.097). The numbers of patient days were associated with lower rates of PI/100 patient days in both IW (IW-2017: IRR for 10 medication analyses: 0.966, 95% CI 0.952–0.980, *p* < 0.0001 and IW-2019: IRR for 10 medication analyses: 0.960, 95% CI 0.946–0.973, *p* < 0.0001). Furthermore, there was an association of the absolute number of documented PI with higher rates of PI/100 patient days (IRR per 10 PI: 1.113, 95% CI [1.066–1.163], *p* < 0.0001) as well as the professional experience as hospital pharmacist (IRR per year: 1.054, 95%CI [1.014–1.095], *p* = 0.007).

### Pharmacists’ interventions

As it is possible to choose multiple reasons for PI in DokuPIK, the numbers of documented reasons were 2701 for 2282 PI (IW-2017) and 2691 reasons for 2578 PI (IW-2019), respectively, corresponding to more documented reasons per PI in IW-2017 as compared to IW-2019 (1.18 vs. 1.04, *p* < 0.001).

While in both IW reasons from the main categories “drugs” and “dose” were selected most frequently, the distribution of reasons between IW-2017 and IW-2019 differed considerably with significant changes in 15 of 26 categories. The largest differences were found in the categories DR 2 ((clear) indication, but no drug prescribed; 8.9 vs 14.7%), DR 8 (inappropriate or not most suitable drug formulation in terms of indication; 1.5 vs. 4.3%), O 3 (procurement/costs; 1.4 vs. 4.4%) and D1 (failure to adjust dose for organ dysfunction; 4,95 vs. 7.0%) being significantly more prevalent in IW-2019, while DR 7 (transcription error; 6.0 vs 1.7%), DR 11 (prescription/documentation incomplete/incorrect; 12.2 vs. 5.2%) and I (interaction; 11.3 vs 6.9%) were noticeable less frequent (details are provided in Table 2). In both IW drugs from the therapeutic main groups J01 (antibacterials for systemic use, 12.7 and 16.8%, respectively) and B01 (antithrombotic agents, 8.5 and 11.3%, respectively) were most frequently involved in PI.

**Table 2:** Reasons for PI in IW-2017 and IW-2019

Code	Reason for PI	IW-2017 (n = 2282)		IW-2019 (n = 2578)		*p*-value
ADM 1	Request/query concerning administration/compatibility	19	0.83%	21	0.81%	0.945
ADM 2	Administration (route)	38	1.67%	34	1.32%	0.319
ADM 3	Administration (duration)	14	0.61%	25	0.97%	0.165
ADM 4	Incompatibility or incorrect preparation or reconstitution	2	0.09%	5	0.19%	0.329
**ADM total**		**73**	**3.20%**	**85**	**3.30%**	
**ADR**	**Adverse drug reaction**	**79**	**3.46%**	**45**	**1.75%**	** < 0.001**
**CI**	**Contraindication**	**77**	**3.37%**	**96**	**3.72%**	0.512
D 1	Failure to adjust dose for organ dysfunction	113	4.95%	181	7.02%	**0.003**
D 2	(Inappropriate) dose	268	11.74%	249	9.66%	**0.019**
D 3	(Inappropriate) administration interval	120	5.26%	157	6.09%	0.212
D 4	TDM not performed or not considered	94	4.12%	124	4.81%	0.246
**D total**		**595**	**26.07%**	**711**	**27.58%**	
DR 1	(Clear) indication not (or no longer) given	291	12.75%	271	10.51%	**0.015**
DR 2	(Clear) indication, but no drug prescribed	202	8.85%	379	14.70%	**< 0.001**
DR 3	Drug allergy or medical history not considered	26	1.14%	9	0.35%	**0.010**
DR 4	Double prescription	82	3.59%	74	2.87%	0.154
DR 5	Dispensing error on the ward	0	0.00%	4	0.16%	0.060
DR 6	Generic/therapeutic substitution	92	4.03%	57	2.21%	** < 0.001**
DR 7	Transcription error	137	6.00%	43	1.67%	** < 0.001**
DR 8	Inappropriate or not most suitable drug formulation in terms of indication	35	1.53%	111	4.31%	** < 0.001**
DR 9	Inappropriately or not most suitable drug in terms of costs	14	0.61%	8	0.31%	0.116
DR 10	Inappropriate or not most suitable drug in terms of indication	94	4.12%	145	5.62%	**0.015**
DR 11	Prescription/documentation incomplete/incorrect	279	12.23%	133	5.16%	** < 0.001**
**DR total**		**1252**	**54.86%**	**1234**	**47.87%**	
**I**	**Interaction**	**257**	**11.26%**	**177**	**6.87%**	**< 0.001**
O 1	Advisory service/drug choice	153	6.70%	112	4.34%	** < 0.001**
O 2	Advisory service/drug dose	161	7.06%	94	3.65%	** < 0.001**
O 3	Procurement/costs	32	1.40%	114	4.42%	** < 0.001**
O 4	Failure to discontinue relevant drugs pre-/perioperatively	10	0.44%	15	0.58%	0.485
O 5	Patient counselling or education	12	0.53%	8	0.31%	0.241
**O total**		**368**	**16.13%**	**343**	**13.30%**	

Bold values in last column reflects statistically significant values

*ADM* administration, *ADR* adverse drug reaction, *D* dose, *DR* drug, *I* interaction, *O* other). As multiple choices of reasons per PI were possible, the sum of reasons is higher than the number of PI. Percentages are calculated on the number of PI

**Fig. 2:**
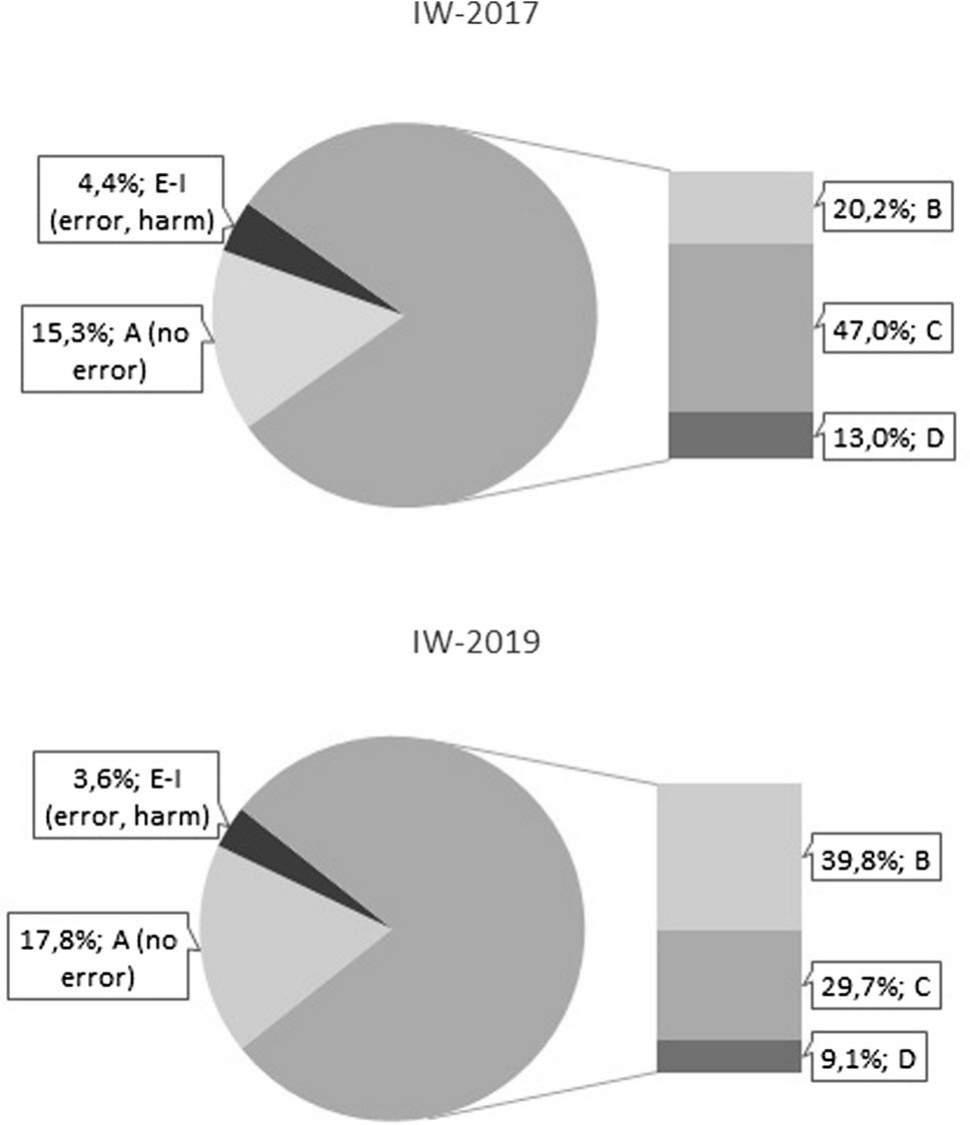
Classification of medication errors according to NCC-MERP. (A: no error, B: an error occurred but the error did not reach the patient, C: an error occurred that reached the patient, but did not cause patient harm, D: an error occurred that reached the patient and required monitoring to confirm that it resulted in no harm to the patient and/or required intervention to preclude harm, E: an error occurred that may have contributed to or resulted in temporary harm to the patient and required intervention, F: An error occurred that may have contributed to or resulted in temporary harm to the patient and required initial or prolonged hospitalization, G An error occurred that may have contributed to or resulted in permanent patient harm, H An error occurred that required intervention necessary to sustain life, I:an error occurred that may have contributed to or resulted in the patient’s death)

In 1909 (83.7%) PI of IW-2017 and 2394 (92.9%) PI of IW-2019 information about the classification of the underlying ME according to NCC MERP was available. The vast majority of medication errors in both IW (80.3 and 78.6%, respectively) were categorized as “error, no harm” (NCC-MERP B-D), while about one in 25 ME (4.4% vs 3.6%) was graded as “error, harm” (NCC-MERP E–I). As shown in Fig. 2, there is a noticeable shift between the two IW (*p* < 0.001): in IW-2017 nearly half of the ME were classified as NCC MERP C (an error occurred that reached the patient, but did not cause patient harm), while in IW-2019 this proportion decreased to about one third. Instead NCC MERP B (an error occurred but the error did not reach the patient) nearly doubled from 20.2 to 39.8%.

Excluding those PI in which only information was provided to either physicians or nurses (10.7 and 8.1%, respectively), the overall acceptance rates of PI significantly increased from 79.9% (IW-2017) to 88.4% (IW-2019), while the proportion of PI with unknown outcome decreased from 14.9 to 6.2% (*p* < 0.001). Proposed interventions that had not been implemented either due to risk–benefit assessment (3.5 vs. 3.7%) or due to proposal rejection (1.0 vs. 1.2%) both stayed stable at a low level (see Table 3).

**Table 3:** Acceptance of PI (PI in which only information was provided to either physicians or nurses (10.7 and 8.1%, respectively) were not included)

	IW-2017 (%)		IW-2019 (%)	
Intervention proposed and implemented	1628	79.9	2096	88.4
Intervention proposed, not implemented (proposal rejection)	21	1.0	28	1.2
Intervention proposed, not implemented (risk–benefit assessment)	72	3.5	88	3.7
Intervention proposed, outcome not known	303	14.9	146	6.2
Problem not solved	14	0.7	12	0.5

## Discussion

Clinical pharmacists in German hospitals importantly contribute to optimise patients’ medication providing routine proactive medication management services. While in IW-2017 in every fourth patient day a pharmacist intervened, in IW-2019 this proportion increased to more than every third patient day. The noticeable increase in ME identified by CP before reaching the patient shows that PI become more proactive instead of reactive and patients are protected from ME early in the medication use process. This may be a positive effect of the increasing proportion of CPS performed on a daily basis. This deeper involvement in direct patient care might also reflect the observed changes towards more complex interventions in IW-2019, as expressed by the increase or reasons for PI regarding drug choice and dose adjustment as compared to the decrease reasons, that can easily be reduced or even avoided using electronic support (e.g. transcription errors, incomplete prescriptions, interactions).

As expected, the intervention rates are higher with rising professional experience and the absolute number of documented PI. However, we observed a small negative effect of performed medication analyses on the intervention rates in both IW. This effect might be explained on the one hand by the increasing use of computerised physician order entry (CPOE) with clinical decision support systems (CDSS) that are known to reduce prescribing errors [15, 16] and on the other hand by learning effects as a result of regular interdisciplinary exchange and training and education activities by clinical pharmacists. Taken together, we are confident that the intervention rates are a robust measure, considering the wide range of patient days and PI per participant. The high numbers of patient days reported by some pharmacists can be explained by medication validation within a completely digital closed loop medication management.

There are many studies about the impact of CPS, and especially for PI to reduce DRPs, ME or adverse drug events (ADE) [2, 17–20]. However, comparability is limited between these studies due to different definitions and methods and the lack of a standard denominator. Preventable ADE have been published to range between 4 to 26.5 per 1000 patient days in different settings [4, 21, 22]. With regard to PI, pharmacist validation of medication prescriptions in a French university teaching hospital resulted in a PI rate of 812 per 62,341 medication orders (1.3 per 100 medication orders) [23], while a mean rate of 4.5 PI per day and ward was determined in the setting of closed-loop medication management with pharmacist validation in a German university hospital [24].

As compared to the MEDAP study, where 62 pharmacists in the United States (in- and outpatient care) submitted 924 datasets on ME and related PI during a 2-weeks period [25], considerably more datasets have been provided in our study by similar numbers of pharmacists during a one-week period (2282 PI by 62 pharmacists and 2587 by 52 pharmacists, respectively). Regarding the classification of ME according to NCC-MERP, our results are in good accordance to those of the MEDAP study, with the vast majority of ME not resulting in patient harm (more than 95%). In another study, PI for community hospital inpatients were recorded and classified during a 14-day period from pharmacists of 15 organisations in the United Kingdom. In this study, mainly in rehabilitation patients, a PI was done in one of three charts for one or more medications with a total of 2758 PI in 4077 medication charts (63 PI per 100 medication charts), with two thirds being classified as prescribing errors. This rate markedly exceeds our rates of 27.5 (IW-2017) and 38.5 PI/100 patient days (IW-2019).

The acceptance rate in IW-2019 (88.4%) is comparable to other published data [14, 19, 22, 24–26]. Our results are especially in good accordance with the latest French national data of 34 522 PIs registered by 201 pharmacists working in 59 hospitals over a 30-months period, where an acceptance rate of 86% was reported with a significant association to the level of pharmacist integration in the ward [27]. The lower rate in IW-2017 (77.9%) might be explainable by the relatively high proportion of PI with unknown acceptance (14.9%). This high rate might result from the much lower frequency of CPS (about 40% less than daily in IW-2017 as compared to about 13% in IW-2019), which makes it more difficult to completely track the acceptance of the PI. This is especially true in those cases, when the PI is not immediately accepted, for example if the junior physician on the ward needs to discuss the PI with a senior physician, patients have only short hospital stays and/or no electronic patient record is available.

The high numbers of both participating pharmacists and documented PI are clearly strengths of our study. Thus, we are confident, that our data represent a cross section of routine CPS in Germany. The fact that the majority of participants work in university hospitals reflects the general situation in German hospitals regarding the staffing levels of clinical pharmacists in different types of hospitals, with a median of 2.7 full-time equivalents per hospital for university hospitals and 1.0 for maximum care and general hospitals, respectively [10]. Furthermore, the willingness to participate in scientific studies might be slightly higher for employees from university hospitals. Another advantage of our study is that we collected data longitudinally using the same methodology, to detect developments in the extent and characteristics of CPS. To the best of our knowledge, no comparable studies have been conducted before.

Possible limitations of our study are the heterogeneity of working environment of the participants concerning the frequency and manner of CPS, paper-based versus electronic prescribing (including CPOE-CDSS), although the PROTECTED-UK study did not show a correlation between the presence of electronic prescribing in critical care and intervention rates [5] and/or traditional supply of whole drug packages with distribution by the nursing staff versus patient-individual logistic provided in the pharmacy. On the other hand, using this cross-sectional approach, we gain insight into the daily routine of CPS in German hospitals. The relatively short time periods of five working days for each period as the basis for our study, might not fully represent everyday work, however we aimed to obtain a data collection of PI as complete as possible for a given time period. To have as many participants as possible and to obtain comparable data to similar studies from other countries we decided to perform our data collections on a five working day basis. As the participation in the DokuPIK IW was voluntary, it might mean that data might have been provided form more engaged pharmacists and therefore the results might not be fully generalizable to ‘pharmacy in Germany’ broadly. Nevertheless, the comparison between the two IW is supposed to provide reasonable reliable results.

In Germany the development of quality and/or key performance indicators for CPS in hospitals as part of the current guideline on inpatient care by hospital pharmacists [28] is about to be completed, based on the publications from other countries [29–31]. Therefore, the data from our study serve as valuable basis for quality indicators regarding the intervention rates of clinical pharmacists in Germany. In this context, ADKA DokuPIK as a structured and validated documentation tool, is intended to further serve as a national documentation platform for quality indicators and is going be developed further to fulfil these requirements.

## Conclusion

In a setting, where clinical pharmacists are closely involved in drug prescribing and medication management as members of the interdisciplinary team on the ward, DRP can be detected by clinical pharmacists at an early stage, before they can cause harm to the patient in most cases. In the future, an additional instrument to rate the relevance of the PI for the patient will be implemented into DokuPIK 2.0 in order to further strengthen the evidence for the importance of CPS.
